# A Boarding Hospital

**Published:** 1902-01

**Authors:** 


### A Boarding Hospital.

THE attention of physicians and others interested is invited to the fact that Miss M. S. Campbell, late of Welcome Hall, and Mrs. A. G. Goodwin, late of the Buffalo General Hospital, have opened a house at No. 50 Allen Street, where out-of-town patients can be received and cared for while under the treatment of Buffalo physicians. Diets will be especially provided for and vapor baths, and the like, included in the price of board and room, which will be from $20 to $25 a week. Friends of patients in hospitals can also be received when there are vacant rooms. Board without treatment will be $12 a week, or for two in one room, $10. Should a friend desire to remain with the patient, the extra charge will be $10 a week. Orders will also be received for diets, such as broths, beef juice, modified milk, wine jelly, and the like. These will be prepared as ordered and delivered at the home of the patient.

The JOURNAL is pleased to direct the attention of physicians in this vicinity to this institution, which may be called with appropriateness a boarding hospital. The women who are conducting it are well known and perfectly trustworthy. Physicians, patients and their friends may feel sure of courteous and faithful attention.

